# Supplementary material for: Open source laser-scanning photostimulation for neuroscience with Zapit
Source: eLife. 2026 Oct 26;15:RP111872. doi: 10.7554/eLife.111872 (PMC13707375; doi:10.7554/eLife.111872)
Supplement: Supplementary file 1 [file elife-111872-mdarchecklist1.pdf]

MDAR Checklist for Authors

Lohse, Gauld, Quigley, Masood et al. — "Open source laser-scanning photostimulation for neuroscience with Zapit"

Materials

Item	Where provided (section / figure legend) and response	n/a
Newly created materials	Data Availability Statement: all hardware designs (CAD, parts lists) and software are openly available at github.com/Zapit-Optostim (software LGPL-2.1; CAD BSD-3-Clause). Build and usage documentation at zapit.swcmicroscopy.com (Results, Figure 1D). No restrictions.	
Antibodies		n/a
DNA and RNA sequences		n/a
Cell materials		n/a
Experimental animals	Methods, "Mice": Mus musculus, VGAT-ChR2-YFP (Jackson Laboratories; JAX stock #014548, RRID:IMSR_JAX:014548).	
Plants and microbes		n/a
Human research participants		n/a

Design

Item	Where provided (section / figure legend) and response	n/a
Study protocol	Not pre-registered.	n/a
Laboratory protocol	Surgical procedure: Methods, "Surgical procedures" and Figure 4 - figure supplement 1. Hardware build, calibration and use: zapit.swcmicroscopy.com. Behavioural protocols cited in Methods (Gauld et al. 2024, 2025; Orsolic et al. 2021; International Brain Laboratory 2021, 2025).	
Sample size determination	Not done. No formal power analysis; this is a methods/tool paper and the in vivo experiments are validation experiments. Sample sizes are given in the Methods and in the Figure 8 and 9 legends.	
Randomisation	Methods: stimulation site, epoch and trial type were randomised trial by trial (whisker task: laser on 50%/33% of trials; visual change detection: site and power randomised; visual discrimination: 50-70% of trials, site pair drawn at random). Mice were not randomised to groups because all experiments are within-subject.	
Blinding	Experimenters were not blinded.	
Inclusion/exclusion criteria	Methods: ephys unit inclusion criteria ("Electrophysiological recordings"); visual change detection sessions included only if performance on the largest change was >80% hits; visual discrimination mice moved to the Zapit rig after reaching the IBL "training 2b" criterion.	
Sample definition and in-laboratory replication	Figure 8 legend and Methods: ephys, n = 117 M2 and n = 23 V1 units from 1 mouse (Fig 8D); n = 354 M2 units from 2 mice (Fig 8E). Figure 9 legend and Methods: whisker mapping, 76 sessions from 4 mice; ALM choice bias, 11 sessions from 3 mice; visual change detection, 5 mice. Units and sessions are nested within biological replicates (mice). Hardware characterisation (Figs 6, 7, Fig 7 - figure supplement 1) uses technical replicates (trials/ measurements), with n given in the legends.	
Ethics	Methods, "Mice": UK Animals (Scientific Procedures) Act 1986, PPL PD867676F, approved by the Sainsbury Wellcome Centre Animal Welfare Ethical Review Body.	
Dual Use Research of Concern (DURC)		n/a

Analysis

Item	Where provided (section / figure legend) and response	n/a

Attrition	Exclusion criteria were set in advance of analysis (Methods): ephys units with poor isolation or drift were excluded; visual change detection sessions below the performance criterion were excluded; trials with early licks or wheel movement were aborted. No animals were excluded [confirm].	
Statistics	Methods, task subsections: GLMMs with a mouse random effect, compared by 20-fold cross-validation and a paired t-test on log-likelihoods; Wilcoxon rank-sum test for reaction times; Bonferroni-Holm correction across 17 sites; paired t-tests across mice (visual change detection). Mean and 95% CI in the figure legends.	
Data availability	Data Availability Statement: processed data for all quantitative figures are at github.com/Zapit-Optostim/Zapit-eLife (MIT licence), with one folder per figure. Figure 8 data are reanalysed from Gauld et al. 2025 (cited). Figure 9C (visual discrimination) data: available on request. Figures 1-5 are schematics or screenshots with no data.	
Code availability	Zapit software at github.com/Zapit-Optostim (LGPL-2.1)	

Reporting

Item	Where provided (section / figure legend) and response	n/a
Adherence to community standards	No specific reporting guideline followed beyond MDAR.	
