# Supplementary material for: Open source laser-scanning photostimulation for neuroscience with Zapit
Source: eLife. 2026 Oct 26;15:RP111872. doi: 10.7554/eLife.111872 (PMC13707375; doi:10.7554/eLife.111872)
Supplement: Peer review history

# Editor report: eLife Assessment

**DOI:** 10.7554/eLife.111872.3.sa0

**Authors:** John R Huguenard

**Affiliations:** Stanford University School of Medicine United States

**Keywords:** Compelling, Important

This work presents a software and hardware suite for targeted photostimulation that can be used in vivo. The package is a well-designed and documented hardware/software suite with a comprehensive build guide. This tool will likely promote **important** neuroscience advances through targeted real-time perturbation of the cerebral cortex. Overall, this manuscript makes a **compelling** case on how to design and make available power tools for the research community.

# Referee report: Reviewer #1 (Public review)

**DOI:** 10.7554/eLife.111872.3.sa1

[Editors' note: this version has been assessed by the Reviewing Editor without further input from the original reviewers. The authors have responded critically to all issues raised in the initial round of reviews.]

Lohse et al. describe an open-source system for laser scanning photostimulation (LSPS) in head-fixed animals. Although similar systems have been developed and used by different groups, Zapit provides an open-source solution requiring few custom parts and minimal coding. This tool can clearly facilitate and speed the adoption of LSPS, particularly for the increasingly used purpose of mapping the effects of focal cortical silencing during behavior. Other potential uses include mapping optogenetically evoked movements and selectively activating genetically labeled neuronal subtypes of interest in the cortex. The design is well thought through, and the presentation is mostly clear and well written.

In general, the more modular such a system is, the better, in terms of compatibility with existing hardware and software that potential users may already have purchased - laser, galvo, and camera in particular.

# Referee report: Reviewer #2 (Public review)

**DOI:** 10.7554/eLife.111872.3.sa2

Summary:

In this work, Lohse and colleagues develop a system for doing targeted photostimulation in mouse cortex. The system uses a camera image to target laser stimulation to stereotactically defined locations in mouse dorsal cortex.

Strengths:

The hardware is well designed, and the software is well documented and supported. The build guide and well-documented software package should allow for simple implementation of the technology. Without a doubt, this is a valuable community resource for the circuit neuroscience field.

Weaknesses:

No weaknesses were identified by this reviewer.

# Referee report: Reviewer #3 (Public review)

**DOI:** 10.7554/eLife.111872.3.sa3

Zappit is an open-source implementation of arbitrary-access laser-scanning optogenetics for manipulation of neuronal activity in mice. As the method requires expertise ranging from optics, hardware control and programming, the authors make the point that this powerful strategy is underutilized in the field, and put forward a well-documented modular hardware and software platform aligned to the Allen Mouse Brain Atlas aimed at enabling the larger scientific community to use this approach (democratizing) for controlling cortical activity during behavior in mice.

The authors favor a galvanometric approach to laser targeting. The system is inexpensive, easy to build, well-documented and user friendly (Matlab based GUI and GitHub repository). The photo-stimulation laser is directed into an X-Y galvo scanner targeted to the specimen using a dichroic mirror and focused on the sample using a Plössl lens as scan lens which is also used as an objective. The scan lens/objective images the specimen onto a camera via tube lens (also a Plössl lens) in a 0.5X magnification ensuring to fit the extent of the mouse brain onto the camera sensor (USB-3 Basler acA120-40um).

The authors report short and reproducible onsite time (~ 0.5 ms) and block (mask) the stimulation source using the laser analog control (~0.5 ms). The system is reliable, aiming at up to 20 stimulation sites per sequence considered as quasi-simultaneous (10 ms). They minimize rebound by gentle ramping down of stimulation over 250 ms.

The system is fast to calibrate by mapping scanner positions to pixel space in the camera space and mapping stereotaxic coordinate onto the image of the exposed skull. The theoretical x-y PSF is 70 µm (measured ~90µm) while the authors make the point that due to scattering the photo-stimulation spot size (lateral extent) is about 1 mm in diameter. This is what they also observe in electrophysiological recordings using silicon probes. The effective radius of inactivation depends on laser power, but was about 1 mm for laser powers (1-2-4 mW) on which the authors observed significant behavioral perturbations - in several tasks: (1) a delayed response somatosensory discrimination, (2) a visual detection task assessing changes in temporal frequency of a drifting visual stimulus; and (3) a visual discrimination (International Brain Laboratory task) in which mice were tasked to report the location of visual stimuli by turning a wheel. As proof of principle, the authors used a photo-stimulation set composed of 52 bilateral sites positioned at 0.5 mm interval covering a large network of frontal, motor and somatosensory cortical areas. Indeed, photo-inhibition of frontal motor cortex sites produced robust increases in reaction time. In contrast, stimulation at other motor and somatosensory sites produced modest, but significant decreases in reaction times.

While the approach is not novel, it does serve the need of better disseminating this technique in the research community. Overall, the Zappit is well-documented and easy to build and use, and will have impact in increasing robust use of site directed photo-stimulation (exciting/inhibiting ensembles of neurons at particular ~1 mm size regions of interests across the dorsal surface of the brain). The authors also note that the axial resolution is ~1.5 mm.

# Author comment: Author response

**DOI:** 10.7554/eLife.111872.3.sa4

**Authors:** Michael Lohse, Oliver M Gauld, Maja T Quigley, Ainiah Masood, Chaofei Bao, Jingjie Li, Nathaniel Miska, Gerion Nabbefeld, Quentin Pajot-Moric, Simon Townsend, Peter Vincent, Angela Y Yang, Nikolaos Zervogiannis, Athena Akrami, Chunyu A Duan, Jeffrey Erlich, Sonja B Hofer, Thomas D Mrsic-Flogel, Philip Coen, Robert AA Campbell

**Affiliations:** Sainsbury Wellcome Centre, University College London London United Kingdom; Sainsbury Wellcome Centre, University College London London United Kingdom; University of Cambridge Cambridge United Kingdom; University College London London United Kingdom; Sainsbury Wellcome Centre, University College London London United Kingdom; Sainsbury Wellcome Centre, University College London London United Kingdom; Sainsbury Wellcome Centre, University College London London United Kingdom; University College London London United Kingdom; Sainsbury Wellcome Centre, University College London London United Kingdom; Sainsbury Wellcome Centre, University College London London United Kingdom; Sainsbury Wellcome Centre, University College London London United Kingdom; Sainsbury Wellcome Centre, University College London London United Kingdom; Sainsbury Wellcome Centre, University College London London United Kingdom; Sainsbury Wellcome Centre, University College London London United Kingdom; University College London London United Kingdom; Sainsbury Wellcome Centre, University College London London United Kingdom; Sainsbury Wellcome Centre, University College London London United Kingdom; Sainsbury Wellcome Centre, University College London London United Kingdom; University College London London United Kingdom; Sainsbury Wellcome Centre, University College London London United Kingdom

The following is the authors’ response to the original reviews.

We thank the reviewers for their thoughtful comments on the manuscript. In response to their suggestions, we have:

Improved hardware calibration flexibility and documentation (Rev 1).

Clarified the optical specifications of the system, including axial resolution and working distance (Rev 1).

Updated Figure 2 and Figure S1 (Revs 1 and 2).

Corrected typographical errors and clarified terminology throughout (Revs 1 and 2). In addition, we have a new Zapit release (v1.0.4, which includes release notes), that contains many improvements and bug-fixes including suggestions from reviewers.

**Public Reviews:**

**Reviewer 1 (Public review):**

Lohse et al. describe an open-source system for laser scanning photostimulation (LSPS) in head-fixed animals. Although similar systems have been developed and used by different groups, Zapit provides an open-source solution requiring few custom parts and minimal coding. This tool can clearly facilitate and speed the adoption of LSPS, particularly for the increasingly used purpose of mapping the effects of focal cortical silencing during behavior. Other potential uses include mapping optogenetically evoked movements and selectively activating genetically labeled neuronal subtypes of interest in the cortex. The design is well thought through, and the presentation is mostly clear and well written.

In general, the more modular such a system is, the better, in terms of compatibility with existing hardware and software that potential users may already have purchased - laser, galvo, and camera in particular. The system has struck a reasonable balance between allowing modularity and providing an integrated complete package, but even more flexibility would be welcome for potential users looking to cut costs, as would clearer presentation of such flexibility as already exists.

Comments and suggestions are mostly minor, as follows.

We thank the reviewer for their assessment of the manuscript, particularly the reference to finding the balance between modularity and an integrated package.

(1) Command signals

How is the relationship between analog voltage commands and laser power determined? Is this assumed (or required) to be linear (as Figure 7F implies)? Usability and modularity would be improved by an option to measure or provide a calibration curve for systems with a nonlinear mapping between command voltage and laser power.

We thank the reviewer for this suggestion. Zapit uses a linear calibration by default, which works well for high-quality diode lasers with built-in power control. For users with EOMs or AOMs we have now implemented a feature that allows creation of the appropritate sigmoid calibration curve. This is in Zapit version 1.0.4 and the process for generating the calibration curve is documented on our GitBook doc site. and the commit containing most of the changes is here. We also include a third order polynomial fit, which we hope will help users of some cheaper lasers where the control function has non-linearities. The appropriate non-linear fit is chosen automatically. We describe this in the legend and main text associated with Fig. 7F.

For the grid calibration step, how is the initial mapping from galvo voltage commands to image position determined? Presumably, some sort of initial guess or calculation based on the hardware specifications is needed for the grid calibration to be feasible. Also, how are the number of grid lines and the distance between them determined?

The number of grid lines and their spacing are set via GUI options. The initial galvo-toimage mapping uses a field-centred affine guess based on the user’s "scanners.voltsPerPixel" setting, and the setup process is described in the user guide. The software ships with a suitable default gain value, which is unlikely to require modification. Invert flags for X and Y are also provided. Once beam locations are recorded, a similarity transform is fitted for residual offset, rotation, and scale. The latest Zapit release includes bug-fixes associated with the centering of the initial calibration point grid in the field of view.

Why is the mapping between analog outputs and hardware (galvos, laser, masking light) fixed? This would be trivial to make configurable and allow labs with existing setups to adopt Zapit without rewiring existing hardware.

We kept the mapping fixed for simplicity in both the build instructions and the code. Zapit will require a dedicated DAQ so we do not anticipate re-wiring is a hurdle. Nonetheles, the code is open-source and such a change is possible: the settings file would need to be augmented and the functions that write the analog output waveforms modified.

(2) Laser and optics

In Figure 1, the authors should consider explaining the scanning principle schematically, i.e., depicting how tilting of the scan mirrors translates via the scan lens into beam displacement in the specimen plane. Perhaps Zemax can be used for accurate rendering.

This suggestion mirrors our own thought process, but we opted not to add a ray-tracing rendering to Figure 1, as doing so comprehensively would require illustrating additional optical principles that would detract from accessibility. However, building on the reviewers recommendation, we now provide references explaining the underlying scanning principles for interested readers (Schottdorf et al. 2025, referenced in Fig. 2). The relationship between scanner angle and beam position is also shown diagrammatically in Figure 7.

Since the unexpanded beam greatly under-fills the back aperture of the lens, the z resolution is presumably terrible - which is good! That is, for the purposes of LSPS, this advantageously avoids focus-dependent effects, which might otherwise arise due to (e.g.) skull curvature. The authors should consider pointing this out, as well as providing an estimate of the z resolution.

This is an excellent point. With our specifications (0.8 mm beam diameter, 473 nm wavelength, 200 mm focal length objective), the effective NA is approximately 0.002, yielding a Rayleigh range of approximately 37.6 mm. The beam must therefore travel nearly 4 cm from focus before the point-spread function doubles in width, making the system highly insensitive to skull curvature. We have added this calculation and noted its practical advantage in the revised manuscript. (Section 2.2).

What is the working distance?

The Plossl scan/objective lens is housed at the end of the lens tube, giving a working distance of approximately 20 cm for the 200 mm focal length objective. We have added this information to Figure 2.

**Reviewer 2 (Public review):**

Summary:

In this work, Lohse and colleagues develop a system for doing targeted photostimulation in mouse cortex. The system uses a camera image to target laser stimulation to stereotactically defined locations in mouse dorsal cortex.

Strengths:

The hardware is well designed, and the software is well documented and supported. The build guide and well-documented software package should allow for simple implementation of the technology. Without a doubt, this is a valuable community resource for the circuit neuroscience field.

Weaknesses:

No weaknesses were identified by this reviewer.

We thank the reviewer for their positive assessment of the manuscript, and their vision for Zapit as an important community resource.

**Reviewer 3 (Public review):**

Zappit is an open-source implementation of arbitrary-access laser-scanning optogenetics for manipulation of neuronal activity in mice. As the method requires expertise ranging from optics, hardware control and programming, the authors make the point that this powerful strategy is underutilized in the field, and put forward a well-documented modular hardware and software platform aligned to the Allen Mouse Brain Atlas aimed at enabling the larger scientific community to use this approach (democratizing) for controlling cortical activity during behavior in mice.

The authors favor a galvanometric approach to laser targeting. The system is inexpensive, easy to build, well-documented and user friendly (Matlab based GUI and GitHub repository). The photo-stimulation laser is directed into an X-Y galvo scanner targeted to the specimen using a dichroic mirror and focused on the sample using a Plössl lens as scan lens which is also used as an objective. The scan lens/objective images the specimen onto a camera via tube lens (also a Plössl lens) in a 0.5X magnification ensuring to fit the extent of the mouse brain onto the camera sensor (USB-3 Basler acA120-40um).

The authors report short and reproducible onsite time (~ 0.5 ms) and block (mask) the stimulation source using the laser analog control (~0.5 ms). The system is reliable, aiming at up to 20 stimulation sites per sequence considered as quasi-simultaneous (10 ms). They minimize rebound by gentle ramping down of stimulation over 250 ms.

The system is fast to calibrate by mapping scanner positions to pixel space in the camera space and mapping stereotaxic coordinate onto the image of the exposed skull. The theoretical x-y PSF is 70 µm (measured ~90µm) while the authors make the point that due to scattering the photo-stimulation spot size (lateral extent) is about 1 mm in diameter. This is what they also observe in electrophysiological recordings using silicon probes. The effective radius of inactivation depends on laser power, but was about 1 mm for laser powers (1-2-4 mW) on which the authors observed significant behavioral perturbations - in several tasks: (1) a delayed response somatosensory discrimination, (2) a visual detection task assessing changes in temporal frequency of a drifting visual stimulus; and (3) a visual discrimination (International Brain Laboratory task) in which mice were tasked to report the location of visual stimuli by turning a wheel. As proof of principle, the authors used a photo-stimulation set composed of 52 bilateral sites positioned at 0.5 mm interval covering a large network of frontal, motor and somatosensory cortical areas. Indeed, photo-inhibition of frontal motor cortex sites produced robust increases in reaction time. In contrast, stimulation at other motor and somatosensory sites produced modest, but significant decreases in reaction times.

While the approach is not novel, it does serve the need of better disseminating this technique in the research community. Overall, the Zappit is well-documented and easy to build and use, and will have impact in increasing robust use of site directed photo-stimulation (exciting/inhibiting ensembles of neurons at particular ~1 mm size regions of interests across the dorsal surface of the brain). The authors also note that the axial resolution is ~1.5 mm.

We thank the reviewer for their detailed assessment of Zapit.

Concerns & comments:

(1) While the authors argue that it offers the best utility to affordability trade-off - faster than motorized drivers and require much less power than DMDs (100X) and less expensive/easier to use compared to SLMs, in the current form, the manuscript does not clearly list the limitations of the approach. At such, in my opinion, the authors should include side by side comparisons (perhaps as a table). For example, clear statements should be included with respect to comparisons in lateral (x-y), axial (z) spatial resolution, as well as temporal sequential aspect of Zappit and other photo-stimulation techniques involving DMDs or SLMs.

Section 3.2 (Comparison to other approaches) compares the scanner-based approach to related techniques. Whilst this is brief, we believe it is adequate because the resolution is ultimately limited by tissue scattering. Indeed, we demonstrate that the radius of neural inhibition ~10 times larger at 2 mW than the lateral PSF (Figure 8D). The size of a DMD pixel on the brain will likely also be smaller than the excitation area, but it does depend on the imaged size of the DMD on the brain. Since that can vary from system to system, a comparison of even theoretical resolution is not straightforward. In terms of spatial patterning, DMDs and SLMs allow for arbitrary shapes to be created on the brain and we point this out in section 3.2.

(2) Is power really a limitation in terms of the laser sources? Or is this a disadvantage mainly because using less power has beneficial effects on the tissue health? It may be useful to provide metrics of comparisons along these lines between Zappit and DMD-based approaches.

The reviewer highlights an important distinction. Too much laser power can cause phototoxicity, and can make neurons more excitable due to heating. It also results in a larger region of stimulation and increases the chance of off-target effects. However, when activating multiple sites with a galvo-based system like Zapit, dwell time goes down ~linearly as the number of “simultaneous” stimulation sites increases. Therefore, even if the same power at the sample is maintained (and the same risk of phototoxicity), peak power must go up to provide the same average power at each site. For example, stimulating 20 points at 40 Hz requires 10 times the peak power compared to stimulating 2 points at 40 Hz.

The same is true of DMD-based approaches. For example, the Mightex recommend a 1 to 4 W laser to run their Polygon DMD-based photostimulation system over an area the size of the mouse dorsal cortex (personal communication); Kauvar, et al. 2020 used a 5 W laser to cover an area 7 mm across using a Polygon system. The cost of such a laser and the Polygon alone likely exceeds 60,000 USD.

Other than prices and logistics, the laser powers needed at the sample and their duty cycles are essentially the same across approaches and so there are no meaningful comparisons we can provide in this domain. However, based on the reviewer’s comments, we now clarify the effects of heating from high laser powers on neural excitability in the discussion (Section 1.1).

(3) Arbitrary-scanning vs random scanning may be more appropriate to describe to strategy.

We agree with the confusion surrounding “random-scanning” and have chosen the phrase “laser-scanning” rather than “random access”, which is the term used in the original pre-print.

**Reviewer 1 (Recommendations for the authors):**

Consider noting that most other galvo scanner models can probably be used.

We have added: "Other scanners could also be substituted, as can other lasers, lens combinations, and laser wavelengths etc.” (Section 4.3).

The basic version of Zapit requires MATLAB (although alternatives are possible and guidance/code is provided), which is not unreasonable but may limit adoption.

We acknowledge this point. MATLAB is widely available in academic neuroscience laboratories, and we provide a Python-based interface, but a complete conversion is beyond the scope of this manuscript. We hope that the open-source code will be adapted to other languages by the community as needed.

Where laser power is mentioned (e.g., Discussion: "we recommend using 1-2 mW time-averaged light power..."), it is not always clear if this is at the laser or in the specimen plane; clarifications would be helpful.

Thank you, we have clarified throughout that reported laser powers refer to measurements at the specimen plane.

Abstract: "causally manipulating" - the "causal" part is redundant and can be dropped, or replaced with "transient" (more relevant).

Changed to "transiently".

Section 2.2: "The modular and open-source nature of Zapit means that exciting new configurations" - strike "exciting".

Corrected.

Figure 7D - y-axis label text is clipped.

Corrected.

**Reviewer 2 (Recommendations for the authors):**

(1) In Figure S1 - Our paper (Heindorf et al.) is incorrectly listed as not making code available - the "camera controlled laser stimulation" software we used is part of Iris2p (that is freely available on SourceForge and linked as such in the paper). Granted, it's not user-friendly or easy to find in the large Iris2p software package - but it is technically "available".

We apologise for this oversight. We have updated the Figure S1 legend to note that “available” code in this context refers to a dedicated and documented standalone package. We have also added links to both Pinto and the Keller-lab software packages.

(2) In Figure 3: "and THE beam goes"

Corrected.

(3) In Figure 4: "the useR will be prompted"

Corrected.
